# Anemia and Its Connections to Inflammation in Older Adults: A Review

**DOI:** 10.3390/jcm13072049

**Published:** 2024-04-02

**Authors:** Eryk Wacka, Jan Nicikowski, Pawel Jarmuzek, Agnieszka Zembron-Lacny

**Affiliations:** 1 Department of Applied and Clinical Physiology, Collegium Medicum University of Zielona Gora, 65-417 Zielona Gora, Poland; jan.nicikowski@gmail.com (J.N.); a.zembron-lacny@cm.uz.zgora.pl (A.Z.-L.); 2 Department of Neurosurgery and Neurology, Collegium Medicum University of Zielona Gora, 65-417 Zielona Gora, Poland; p.jarmuzek@cm.uz.zgora.pl

**Keywords:** aging, anemia diagnosis, erythropoiesis, geriatric diseases, inflammation, iro deficiency, hypoferremia, oxidative stress

## Abstract

Anemia is a common hematological disorder that affects 12% of the community-dwelling population, 40% of hospitalized patients, and 47% of nursing home residents. Our understanding of the impact of inflammation on iron metabolism and erythropoiesis is still lacking. In older adults, anemia can be divided into nutritional deficiency anemia, bleeding anemia, and unexplained anemia. The last type of anemia might be caused by reduced erythropoietin (EPO) activity, progressive EPO resistance of bone marrow erythroid progenitors, and the chronic subclinical pro-inflammatory state. Overall, one-third of older patients with anemia demonstrate a nutritional deficiency, one-third have a chronic subclinical pro-inflammatory state and chronic kidney disease, and one-third suffer from anemia of unknown etiology. Understanding anemia’s pathophysiology in people aged 65 and over is crucial because it contributes to frailty, falls, cognitive decline, decreased functional ability, and higher mortality risk. Inflammation produces adverse effects on the cells of the hematological system. These effects include iron deficiency (hypoferremia), reduced EPO production, and the elevated phagocytosis of erythrocytes by hepatic and splenic macrophages. Additionally, inflammation causes enhanced eryptosis due to oxidative stress in the circulation. Identifying mechanisms behind age-related inflammation is essential for a better understanding and preventing anemia in older adults.

## 1. Introduction

The world population is rapidly aging, and this demographic shift is expected to continue over the coming decades. This phenomenon is characterized by an increase in both the number and the percentage of older adults worldwide. Currently, 10% of the world population is aged 65 years or older, but this figure is expected to reach 16% by 2050. Developing countries are particularly affected by this trend due to the declining levels of mortality, as reflected in the increased levels of life expectancy at birth [1]. As individuals grow older, their organic functionality naturally declines over time (aging), ultimately resulting in death. Aging is also associated with an increased likelihood of common conditions such as cardiovascular diseases, cancer, diabetes, or neurodegenerative diseases, which, in turn, elevate the risk of mortality [2].

Anemia, a condition that frequently occurs in older patients, has no standard definition. The World Health Organization (WHO) established the diagnostic criteria for anemia, which was defined as a hemoglobin (Hb) level < 13.0 g/dL for men and <12.0 g/dL for women [3]. Since the WHO definition of anemia was established more than five decades ago on the basis of a limited population sample and without proper documentation of the methodology used, understandably, there are now certain restrictions related to these thresholds. Nevertheless, the WHO definition continues to be the standard for anemia classification in older adults, despite suggestions from various studies that the definition be revised. Higher Hb reference values to define anemia were suggested after the analyses of American databases including the National Health and Nutrition Examination Survey III [4] and the Scripps-Kaiser database [5]. The Cardiovascular Health Study [6] identified optimal Hb levels of ≥13.7 g/dL for men and ≥12.6 g/dL for women, which were recorded to be associated with improved survival. The population study by Culleton et al. [7] determined that optimal Hb values of 13.0 to 15.0 g/dL for women and 14.0 to 17.0 g/dL for men could help avoid hospitalization and reduce the risk of mortality in old age. Wouters et al. recommended modifying Hb values to <13.0 g/dL for women over 60 years of age to align with the definition used for men [8].

Age-related, chronic, low-grade inflammation is not only a consequence of increasing chronological age, but also a marker of biological aging, multimorbidity, and mortality risk [9]. Systemic inflammation can significantly exacerbate health status and lead to a decline in overall well-being [10]. As the immune system ages, its ability to effectively respond to and manage inflammation diminishes, which renders the elderly more susceptible to a range of diseases such as anemia [11,12]. Therefore, the objective of this review was to explore the pathophysiological causes of anemia in the elderly, particularly those associated with inflammation, and to elucidate the underlying mechanisms and contributing factors for anemia in this age group.

### Prevalence of Anemia

The prevalence of anemia varies across age groups, genders, and races, and the condition is more common in older individuals, with higher rates observed in men compared to women and in black individuals compared to white ones. However, it is noteworthy that most individuals classified as anemic according to the WHO criteria demonstrated anemia of a mild degree [3].

A systematic review of 34 studies showed that in people aged >65 years, the prevalence of anemia was recorded in 12% of community-dwelling persons, 40% of hospitalized patients, and 47% of nursing home residents [13], with the overall mean prevalence of 17% [14]. The increased prevalence of anemia among nursing home residents was often attributed to poorer health status and the higher occurrence of comorbidities in the elderly residents of these facilities compared to the community-dwelling age-matched population [15]. Insights into the prevalence of anemia across different populations and its findings, based on selected studies, are summarized in Table 1.

The severity of anemia was found to be higher in skilled nursing facilities compared to community-based settings, as revealed in a survey of five such facilities where a hemoglobin level ≤ 10 g/dL was detected in 11.4% of the residents [19]. In hospitalized patients aged ≥65 years, the prevalence of anemia reached up to 48%, with 65% of patients exhibiting mild (Hb > 10 g/dL) to moderate (Hb 8–10 g/dL) anemia [20]. Interestingly, it was observed that the recognition and investigation of anemia were rarely undertaken [21]. These findings highlight the increased severity of anemia in skilled nursing facilities and the need to raise the awareness of the staff and the management of this condition in both health care settings.

It is evident that the analyzed issue differs depending on the geographical location and the economic status of various countries.

The identification of the putative factors underlying anemia of inflammation in older adults poses a considerable challenge as this age group is affected by a tremendous extent of subclinical and clinical morbidities as well as an age-related increase in the levels of proinflammatory cytokines. It is therefore hardly surprising that nearly a fifth of anemia cases (19.7%) in older adults have been classified as anemia of inflammation, also known as anemia of chronic disease [1]. However, distinguishing anemia of chronic inflammation from iron deficiency anemia is particularly challenging in older adults due to the comorbid effects of gastrointestinal bleeding and the effects of medications [22]. Serum ferritin levels can still fall within the reference range when both types of anemia are present, which might potentially have led to an overestimation of anemia of chronic inflammation prevalence in the NHANES III study [1] at the expense of iron deficiency anemia. Furthermore, even distinguishing anemia of chronic inflammation from anemia of chronic kidney disease is somewhat tenuous, given the emerging evidence of increased inflammation associated with renal function in older adults without chronic kidney disease [23,24].

## 2. Causes of Anemia in Older Adults

**Figure 1:**
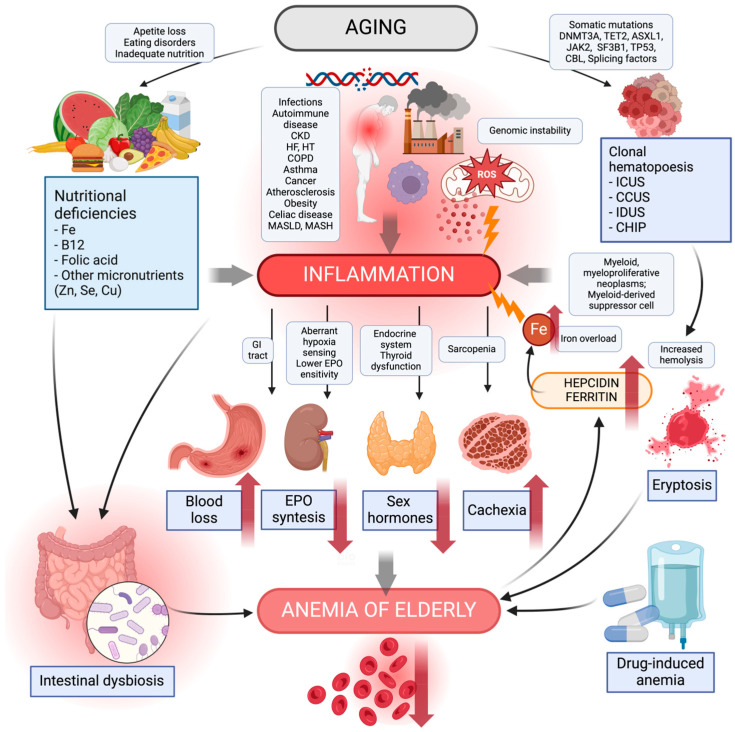
Causes of anemia in the elderly. The diagram shows the main causes of the development of aging-related inflammation that can contribute to anemia in the elderly. Aging processes such as genome instability, reactive oxygen species in the mitochondria, synthesis of pro-inflammatory cytokines, negative environmental factors, and chronic diseases lead to inflammation. Inadequate nutrition, eating disorders, and loss of appetite contribute to an increased risk of nutritional deficiencies—iron, vitamin B12, folic acid, zinc, selenium, and copper. Their deficiency leads to inflammation and modulation of the intestinal microbiota to its detriment, increasing the risk of intestinal dysbiosis. The number of somatic mutations increasing with age can lead to clonal hematopoiesis. This, in turn, increases the incidence of myeloid myeloproliferative neoplasms, myeloid-derived suppressor cells causing inflammation. Clonal hematopoiesis shortens the lifespan and durability of erythrocytes, increasing their risk of hemolysis, the process of eryptosis. Chronic inflammatory processes further contribute to gastrointestinal inflammatory disease and blood loss; a decrease in sensitivity to hypoxia and EPO, thereby causing a reduction in EPO synthesis; endocrine dysfunction causing a decrease in sex hormones; and a decrease in muscle mass to sarcopenia, leading to the risk of cachexia. Pharmacotherapy with drug–drug interactions can produce adverse effects potentially contributing to anemia in older adults. Lately, anemia in the elderly has been reported to cause an increase in hepcidin, a plasma ferritin causing pro-inflammatory iron overload. CCUS, clonal cytopenia of unknown significance; CHIP, clonal hematopoiesis of indeterminate potential; CKD, chronic kidney disease; COPD, chronic obstructive pulmonary disease; EPO, erythropoietin; GI, gastrointestinal; HF, heart failure; HT, hypertensive; ICUS, idiopathic cytopenia of undetermined significance; IDUS, idiopathic dysplasia of undetermined significance; MASLD, metabolic dysfunction-associated steatotic liver disease; MASH, metabolic dysfunction-associated steatohepatitis; ROS, reactive oxygen species. Created with BioRender.com (accessed on 9 March 2024).

The processes responsible for the maintenance of homeostasis diminish with increasing age, and one of these processes involves a decrease in hematopoietic potential. However, there are no adequate hemoglobin reference values below which anemia can be diagnosed in adults aged over 65 years, so the referential range for the general population is still applied [25]. Figure 1 provides a visual representation of the main factors contributing to the development of aging-related inflammation that can further contribute to the development of anemia in the elderly.

Genomic instability is a fundamental cause of the progressive aging process [26]. The factors favoring genome instability include environmental factors [27], the influence of chemicals [28], oxidative stress [29], hypoxia progression with aging [30], and chronic inflammation [31]. An increase in DNA instability results in elevated numbers of somatic mutations in the resulting cells [32]. With age, somatic mutations also affect all cells involved in hematopoiesis, resulting in clonal hematopoiesis [33]. The incidence of progressive clonal hematopoiesis increases with age [34]. The number of clonal abnormalities is correlated with the risk of developing myeloid and myeloproliferative neoplasms including acute myeloid leukemia, myelodysplastic syndromes, myeloproliferative syndromes, and mixed (myelodysplastic-myeloproliferative) syndromes. The list of the above-mentioned diseases also includes anaplastic anemia.

Increased mitochondrial damage in stem cells [35] and impaired mitochondrial function observed in chronic diseases during hematopoiesis [36] are further elements of the hypothesis concerning the etiology of anemia in old age. Accumulating damage to telomeres leads to aging of the mitochondria and the increased production of reactive oxygen species (ROS), which results in generalized hypoxia as a consequence [37,38]. In the longer term, a persistent increase in hypoxia activates systemic compensatory and adaptive mechanisms [39].

## 3. Pathophysiology of Inflammation Causing Anemia in Older Adults

As individuals age, the phenomenon known as inflammaging becomes increasingly prevalent. Inflammaging is characterized by chronic low-grade inflammation, and it is considered a significant contributor to the aging process. The underlying mechanism involves the release of a multitude of inflammatory mediators that are produced in response to tissue damage and stress. The key players in chronic inflammation include a variety of interleukins such as IL-1, IL-1b IL-2, IL-6, IL-8, IL-12, IL-13, IL-15, IL-18, IL-22, and IL-23. The pro-inflammatory activity of these cytokines initiates and amplifies the inflammatory response. Additionally, tumor necrosis factor α (TNFα) and interferon-γ (IFN-γ) are also prominent pro-inflammatory cytokines. Variations in the genetic sequences within the promoter regions of proinflammatory and controlled cytokine genes can influence the processes of inflammaging and vulnerability to age-related diseases [40].

On the other hand, attempts are made by anti-inflammatory cytokines including IL-1Ra, IL-4, IL-10, and transforming growth factor (TGF-β1) to counterbalance the pro-inflammatory response. These cytokines, in turn, are engaged in the suppression of inflammation and they promote a more balanced immune response. Along with the cytokines, a range of other molecules contribute to the complex network of inflammaging. For instance, lipoxin A4 plays the role of a lipid mediator with potent anti-inflammatory properties. Heat shock proteins are also involved in the regulation of inflammation, acting like chaperones that help to protect cells from stress-induced damage [41,42,43]. According to Minciullo et al. [43], inflammaging is a key to our understanding of the aging process, and anti-inflammaging may be one of the secrets of longevity. Therefore, anemia caused by inflammation is an important issue to be tackled more quickly and multidimensionally. 

### 3.1. Iron Restriction (Hypoferremia)

During infection or inflammatory events, hypoferremia occurs quickly with a decrease in plasma iron level and transferrin saturation, which prevents the formation of nontransferrin-bound iron, a powerful trigger for the pathogenicity of Gram-negative bacteria and potentially also other microorganisms [44,45]. Iron consumption by erythropoiesis and the turnover of senescent or damaged erythrocytes by macrophages are the primary factors affected by various inflammatory processes. Therefore, maintaining strict control over iron levels during inflammation is crucial for host defense.

Hepcidin, a 25-amino-acid peptide released by liver cells, circulates in the blood and is expelled in the urine. Hepcidin serves as the primary governing factor for both iron absorption and distribution across different tissues [46,47]. Elevated levels of circulating hepcidin, induced by IL-6, inhibit the release of cellular iron into plasma by binding to the cellular iron efflux channel ferroportin [48]. Ferroportin occurs on the cells that serve as specialized iron managers within the body, and these cells include duodenal enterocytes that are responsible for the absorption of dietary iron, hepatic and splenic macrophages that recycle senescent erythrocytes, hepatocytes that are engaged in iron storage, and placental trophoblasts that facilitate iron transfer to the developing fetus during pregnancy [49]. 

Macrophages play a crucial role in recycling iron from aging red blood cells and once recycled, the iron is released into the bloodstream through ferroportin. Inflammation triggers an increase in hepcidin levels, thereby leading to enhanced internalization and the breakdown of ferroportin [50]. As a result, the release of ferrous iron from key iron transport tissues such as duodenal enterocytes, iron-recycling macrophages, and iron-storing hepatocytes into the bloodstream is reduced. This leads to the accumulation of iron within their cellular ferritin. Subsequently, the continuous utilization of iron by erythropoiesis depletes the extracellular iron pool, which results in low levels of iron and restricted erythropoiesis.

**Figure 2:**
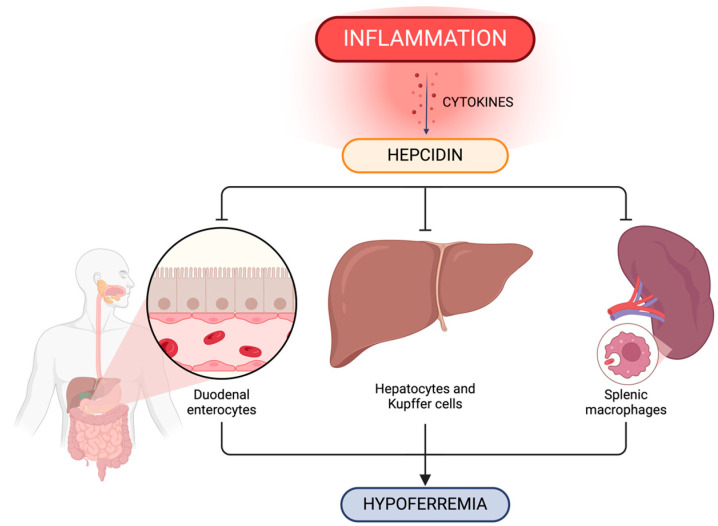
Inflammation impact on the regulation of systemic iron metabolism. Hepcidin plays a crucial role in systemic iron level control via ferroportin concentration in iron-exporting cells including duodenal enterocytes, hepatic and splenic iron-recycling macrophages and hepatocytes. Created with BioRender.com (accessed on 9 March 2024).

Anemia of inflammation is characterized by hypoferremia accompanied by increased plasma ferritin and hepcidin levels, whereas iron-deficiency anemia manifests itself in hypoferremia accompanied by low levels of plasma ferritin and hepcidin. Inflammatory hypoferremia, similar to hypoferremia in systemic iron deficiency, inhibits erythropoiesis, however, the inhibitory effect is detected at a relatively high threshold (transferrin saturation of 15 to 20%), which may suggest a protective function of this mechanism to ensure an adequate iron supply for other tissues such as muscles, the central nervous system, and nonerythroid marrow, which are less affected by decreased plasma iron levels (Figure 2) [51].

Our previous study demonstrated higher hepcidin levels in the group with anemia compared to non-anemic participants [52], which is consistent with other reports, for instance, the Leiden 85-plus Study, which also revealed elevated serum hepcidin levels in older adults with anemia of inflammation. However, the InCHIANTI study did not find an increase in urinary hepcidin levels [48,53]. The available studies also reported differences in hepcidin levels across the compared genders. On average, approximately 50% lower hepcidin levels were observed in premenopausal women than in the age-matched male groups. However, post-menopause hepcidin levels tend to become comparable in both gender groups, which was reported in the Val Borbera study [54] and the Nijmegen Biomedical Studies [55]. 

The impact of hepcidin–ferroportin interaction in iron homeostasis is shown in Figure 2.

### 3.2. Erythropoiesis Suppression

Pro-inflammatory cytokines, especially interferons and TNFα, appear to inhibit the proliferation and differentiation of erythroid progenitor cells, leading to ineffective erythropoiesis as a result [56].

Early inflammatory responses include leukocytosis and the increased production of leukocytes in the marrow, which is manifested by an increased number of myeloid precursors (>4:1 myeloid to erythroid precursors ratio). Inflammatory cytokines such as TNF-α [57] and interferon-γ [58] activate the transcription factor PU.1 and trigger bone marrow reprogramming, which promotes myelopoiesis and lymphopoiesis while suppressing erythropoiesis. Inflammatory cytokines also inhibit the ability of BFU-E to generate more differentiated erythroid cells [59,60].

Another bone marrow-reprogramming mechanism involves inflammation-induced suppression of erythropoietin production, the primary hormone responsible for erythropoiesis. In patients with systemic inflammation, serum erythropoietin levels are lower compared to the individuals with a similar degree of iron-deficiency anemia [61,62]. Inflammation also impairs the responsiveness of erythroid precursors to erythropoietin, as evidenced by increased exogenous erythropoietin requirements in end-stage kidney disease patients with inflammation [63,64]. Resistance to erythropoietin is partly mediated by a decrease in the number of erythropoietin receptors on erythroid progenitors, whose proliferative capacity is therefore reduced, which is a recently discovered effect of hypoferremia [65].

The Klotho enzyme, which is mainly expressed in humans in the kidney and brain by the KL gene, especially its alpha-Klotho variant activated by fibroblast growth factor 23 (FGF23), has been indicated as another potential cause of inflammatory anemia in old age [66,67]. Most of the early as well as current studies have focused on the role of alpha-Klotho in chronic kidney disease (CKD) in elderly populations, and reported an age-related Klotho reduction and association with increased likelihood of anemia [68]. Klotho is involved in hematopoiesis regulation through its impact on the hypoxia-inducible factor (HIF1α) pathway. Its deficiency interferes with hematopoietic stem cell development and erythropoiesis [69]. Klotho has the ability to modulate inflammation and oxidative stress through various mechanisms. As a suppressor gene of aging, Klotho protein expression, among other things, reduces phosphorus, ROS, and slows age-related renal fibrosis [70,71]. Increased Klotho levels could also potentially contribute to inflammation and anemia reduction in the elderly [70].

The production of erythropoietin, a major cytokine that affects the development of red blood cells, is triggered by a mechanism that detects low oxygen levels in anemia conditions. The relationship between the impaired response of hematopoietic stem cells to EPO and the development of anemia was observed in elderly patients [72]. The Baltimore Longitudinal Study on Aging [73] reported that EPO levels increased with age in healthy individuals without anemia, particularly in those without diabetes or hypertension. Conversely, individuals with anemia demonstrated a lower rate of EPO increase, suggesting that anemia is linked to a failure in the normal compensatory rise of EPO levels during aging. Although low EPO levels have specifically been associated with unexplained anemia in the elderly population, the exact cause of the inadequate EPO response is still unknown. Therefore, further studies on larger samples of elderly patients are necessary to confirm these findings [73]. A study by Chencheng et al. [68] suggested that low serum Klotho levels were associated with an increased likelihood of anemia in middle-aged and older adults regardless of kidney disease. 

Overall, the age-dependent impairment of EPO response suggests a progressive resistance of hematopoietic stem cells to EPO as individuals age. The underlying reasons are yet to be determined, however, they could be attributed to impairments in normal EPO-dependent pathways caused by inflammatory cytokines, age-related comorbidities, declines in renal function, or a combination of these factors [74].

### 3.3. Shorter Erythrocyte Lifespan

The available studies on anemia of inflammation have consistently reported a moderate reduction (approximately 2–5%) in the lifespan of red blood cells with a decrease to approximately 90 days. However, a shortened erythrocyte lifespan was also observed in many cases of non-anemic inflammation, which indicates that anemia develops only when the compensatory response of red blood cell production is impaired [75].

The shortened lifespan of erythrocytes during inflammation has been ascribed to macrophage activation triggered by inflammatory cytokines, which results in premature phagocytosis and erythrocyte elimination. Macrocytic anemia and heightened erythrophagocytosis are prominent manifestations observed in macrophage activation syndromes, particularly those associated with systemic juvenile rheumatoid arthritis [76]. Multiple cytokines including interferon-γ and IL-4 have been implicated in macrophage activation for erythrophagocytosis in mouse models [57,77].

Except for rare cases of fulminant hemophagocytic states, erythrophagocytosis in anemia of inflammation exhibits only a mild increase, and the increase could easily be compensated if the production of erythrocytes is unimpaired [78,79].

Furthermore, the inflammatory cascade involves the generation of reactive oxygen and nitrogen species, shaping the intricate interrelationship between inflammation and the behavior of erythrocytes in the circulatory system. The oxidative stress within the vascular bed exerts multifaceted effects on the structural integrity of red blood cells, characterized by lipid peroxidation and the oxidation of membrane skeletal proteins. These biochemical alterations do not only compromise the molecular architecture of red blood cell membranes, but they also have profound implications for their functional properties [80,81,82,83].

The consequence of oxidative stress-induced modifications extends beyond mere structural compromise, significantly impacting the physiological characteristics of red blood cells. Notably, the reduction in osmotic resistance and deformability of red blood cells has emerged as a pivotal outcome of oxidative stress in the vascular microenvironment [84]. The compromised osmotic resistance renders erythrocytes more susceptible to premature removal from circulation as their resilience to environmental challenges diminishes [81,82,85,86].

The intricate relationship between oxidative stress and red blood cell dynamics underscores the accelerated elimination of these cells from the circulatory system. This phenomenon, although crucial in our understanding of broader implications of inflammation-induced alterations, unfortunately remains unexplored in the current discourse. An in-depth exploration of this aspect of oxidative stress is imperative for our thorough comprehension of the intricate mechanisms underpinning anemia in the context of inflammation.

**Figure 3:**
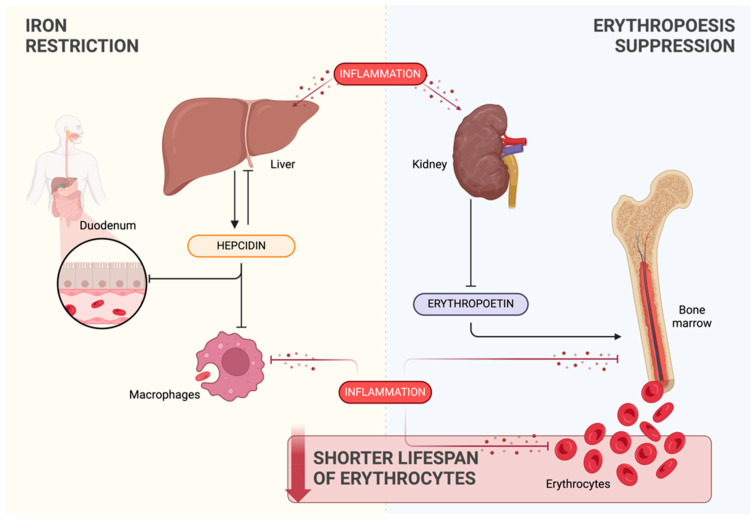
Overview of the processes linked to inflammation-induced anemia: iron restriction (yellow background), erythropoiesis suppression (blue background), and shortened erythrocyte lifespan (red background). Created with BioRender.com (accessed on 9 March 2024).

Figure 3 provides a comprehensive overview of the interconnected processes contributing to inflammation-induced anemia. The schematic representation serves as a visual guide to illustrating the complex dynamics involving the key elements in the pathogenesis of inflammation-associated anemia.

## 4. Diseases Associated with Anemia in Older Adults

Anemia in older adults has a multifactorial cause. Consequently, there are no and major and clear-cut contributors to anemia in the elderly. Overlapping diseases leading to multimorbidity and an increased risk of frailty syndrome make the identification of the causes of anemia even more challenging [87,88]. Nonetheless, the disease entities that are associated with reduced synthesis, disruption of normal hematopoiesis to achieve the desired volume and number of red blood cells and hemoglobin content, can be included in the list of potential anemia-inducing conditions. This section discusses the most common diseases associated with anemia in older adults. As depicted in Table 2, there are numerous common diseases prevalent among the elderly that have the potential to lead to anemia. Understanding these diseases is vital for health care professionals to accurately diagnose and manage anemia in this population.

The commonly reported reasons include the increased risk of nutritional disorders due to an excessive intake or negative balance in dietary supply of energy, nutrients, vitamins, and the inability to replenish the effects of catabolic processes [89]. Cachexia, defined as disease-related malnutrition (DRM) with inflammation in the ESPEN guidelines on definitions and terminology of clinical nutrition [90], has been reported in relevant analyses. 

The process of aging is associated with epigenetic changes that lead to somatic mutations of cells beginning with pluripotent hematopoietic stem cells (PHSCs), which are further growth pathways in hematopoiesis. These alterations result in shorter erythrocyte lifespan and increased eryptosis rate. As a consequence, augmented oxidative stress increases inflammation and the risk of age-related chronic diseases as well as hematopoietic disorders [91,92]. The progressive deterioration of kidney and liver function in elderly populations is a key element in the development of anemia and it involves: firstly, macroscopic changes, mainly a lower mass of the organs and, secondly, microscopic pathological tissue and cells changes such as atherosclerosis of capillaries, atrophy, fibrosis, and collagen deposition. Functional and structural changes in the aging organs increase the probability of erythropoiesis suppression [93,94,95].

### 4.1. Hematopoietic Disorders

Hematopoiesis-regulating mechanisms that involve players such as cytokines, chemokines, hormones, adhesion molecules, and transcription factors occur at each stage of the cell lines, ranging from the process of renewal, through differentiation to maturation of blood cells. However, the process of aging is linked to the impairment to the processes of self-renewal, differentiation, proliferation, and maturation of cells involved in hematopoiesis.

Age-related progressive changes such as epigenetic alterations, genetic instability, telomere shortening, and accumulation of p53 damage have all been reported to affect cellular aging [96,97]. Progressive somatic mutations cause an increase in clonal hematopoiesis of indeterminate potential (CHIP) in an average of 25% of the human population aged over 65 years, with a further increase observed with aging. The most common mutations concern the following genes: DNMT3A, TET2, ASXL1, JAK2, SF3B1, and TP53 [33,98,99,100]. Clonal changes correlate with increased heterogeneity of the cell size indices such as the red cell distribution width (RDW) [100]. These findings have been supported by recent genome-wide association studies. Furthermore, studies have also demonstrated correlations with an increase in inflammatory markers such as CRP, IL-1b, and IL-18, but with a decrease in hemoglobin [98,99,101]. These somatic mutations result in an increased risk of chronic diseases typical of old age, myeloproliferative diseases, and mortality. 

Changes associated with clonal hematopoiesis can also lead to aplastic anemias [102]. Along with changes in the cell population in the hematopoietic lineage, the bone marrow undergoes conversion from hematopoietically active red marrow to hematopoietically inactive yellow bone marrow [103]. A decrease in bone density and disturbances to homeostasis in osteoblast–osteoclast communication are also correlated with an increased risk of anemia in elderly people [104].

Consequently, the balance between new erythrocyte formation and their erythrophagocytosis and hemolysis is disrupted and can lead to a decrease in the number of erythrocytes in the bloodstream. Since oxidative stress is known to affect erythrocyte lifespan, one of the hypotheses for anemia involves the activity of reactive oxygen species on erythrocytes in the course of chronic inflammation in a progressive process of aging.

In the 1980s, Tozzi-Ciancarelli and Fedele showed that the structural properties of erythrocytes differed between older and young adults [105]. The age-related accumulation of defective proteins has further consequences for hematopoiesis, ultimately creating defects in the structure of reticulocytes and erythrocytes such as changes in the spectrin-4.1-actin complex, cytoskeleton structure, glycocalyx, and band protein III, which causes hemolysis [106,107,108,109,110].

### 4.2. Kidney Disease

Chronic kidney disease was defined by the KDIGO (Kidney Disease; Improving Global Outcomes) 2012 Clinical Practice Guideline for the Evaluation and Management of Chronic Kidney Disease as a progressive abnormality of kidney structure and function, a condition leading to end-stage renal disease requiring renal replacement therapy [111]. Uremia progression is accompanied by an increase in chronic inflammation and oxidative stress. The processes of hematopoiesis and eryptosis are therefore disrupted by the accumulation of pro-inflammatory molecules such as CRP, NOS, ROS, IL-1, IL-6, TNF-α, and other inflammatory mediators. Normal nephron functioning deteriorates with age, as shown by a decrease in the glomerular filtration rate (GFR) in the aging population. In a population study of 12,381 Germans, Waas and Schulz [112] recorded a decrease by 1 mL/min/m^2^ estimated GFR (eGFR) per year from the third decade of life. The increased risk of developing progressive nephropathy includes age-related chronic diseases such as hypertension, diabetes, and glomerulonephritis [113]. An epidemiological study by Kovesda et al. [114] recorded 25.3% prevalence of anemia in people with stages 3–5 CKD (eGFR < 60 mL/min/1.73 m^2^). With regard to age groups, anemia was more likely to develop in patients aged ≥75 years, who also demonstrated a significant correlation with lower mean hemoglobin concentrations. According to Stauffer et al. [115], anemia was twice as prevalent in people with CKD as in the general population (15.4% vs. 7.6%, respectively).

The phenomena of polypharmacy and polypragmasy, which belong to the risk factors for kidney disease, are frequently recorded in the elderly [116]. Increased use of drugs available without prescription, primarily, non-steroidal anti-inflammatory drugs (NSAIDs), herbal remedies, and dietary supplements can lead to drug-induced nephrotoxicity [117] with potential consequences being acute kidney injury (AKI), possibly leading to irreversible CKD [118].

Other risk factors of AKI and CKD progression include water–electrolyte disturbances. Older adults belong to the age group at increased risk of disruption of physiological homeostasis such as thirst control disturbances and water–electrolyte imbalance. Dysfunction of central nervous system mechanisms of thirst control results in reduced thirst in response to current water–electrolyte needs [119,120]. These tendencies toward dehydration are the risk factors for hemodynamic weakness, which significantly contributes to the increased risk of kidney damage [121,122].

Erythropoietin, the major regulator in erythropoiesis renewal, is prenatally synthesized in the liver, but hepatic EPO production is switched to the kidneys and taken over perinatally by peritubular interstitial fibroblasts (which belong to renal erythropoietin-producing cells (REPCs)) [123]. In chronic kidney disease, the number of REPCs is reduced due to their differentiation into myofibroblasts, which lose their ability to produce EPO. Therefore, an insufficient number of hypoxia-inducible factor (HIF)-sensitive REPCs in response to hypoxic stimuli causes an EPO decrease and erythropoiesis, leading to the development of anemia [124].

Vlasschaert et al. [125] observed more rapid development of progressive chronic kidney disease and greater severity of anemia in patients with previous CKD and current CHIP.

### 4.3. Hormonal Factors

Hormonal regulation is also affected by age-related changes, which pertains to both genders: menopausal and postmenopausal changes in women and andropausal transition in men have been widely investigated. In both genders, a reduction in circulating estrogen and testosterone plasma levels have been recorded. An age-related decrease in muscle mass due to lower testosterone levels also results in reduced sensitivity of erythropoietin forming [126,127]. Significant correlations between sarcopenia and anemia were detected in population-based studies [128,129,130]. A reduction in erythropoiesis may also result from decreased thyroid hormonal activity, which slows down the anabolic hormone level. Fatigue, weakness, and loss of appetite are among the significant effects of decreased numbers of blood erythrocytes [131]. 

With aging, the endocrine abnormalities decrease the production of hormones and consequently affect red blood cell homeostasis. A potential impact of anabolic hormones (IGF-1, testosterone, TSH, T3, T4) on hepcidin regulation and the expression of progenitor cells involved in hematopoiesis has been recorded [132] The late-onset hypogonadism in andropausal elderly males and hypoestrogenism in postmenopausal elderly females are the hormonal factors that potentially contribute to anemia development. Adequate plasma testosterone levels modulate pro-inflammatory cytokines, mainly IL-6, which ensure appropriate hepcidin levels and proper hematopoietic cell differentiation without clonal cells. These are the elements that favorably affect the hemoglobin and hematocrit levels in older men [133,134]. Reduced testosterone levels correlate with a negative response to erythropoiesis-stimulating factors [135].

It should be noted, however, that in elderly patients with prostate cancer treated with hormone replacement therapy (androgen deprivation), radiotherapy, and brachytherapy procedures, their hemoglobin levels were observed to fall by an average of 1–2.5 g/dL, which should not be directly linked to inflammatory processes and anemia [132,136].

Progenitor cells contain estrogen receptors (ER-α and ER-β) that are influenced by estrogen during differentiation [137]. Zhou and Tseng [138] showed that estrogen regulated erythropoiesis by ROS and NOS modulation on the progenitor erythroid cells, which affected proliferation and differentiation. The research conducted to date has also confirmed the protective cardiovascular effects of estrogens in women as their NOS and ROS-modulating activity produces anti-inflammatory effects [139]. Moreover, estrogen has an erythropoiesis-stimulating effect on bone marrow stem cells, which has been proven to support erythrocyte count and hemoglobin levels in pregnant women [140]. 

Estrogens participate in the estrogen–iron axis through their ability to inhibit hepcidin formation. A decrease in hepcidin results in an increase in iron storage, while an inflammation-induced increase of hepcidin levels, among other factors, negatively affects iron metabolism.

### 4.4. Gastrointestinal Diseases

Gastrointestinal (GI) changes are common in the elderly, with some GI disorders being more prevalent in this age group such as changes in the oral cavity, esophagitis, gastroesophageal reflux disease (GERD), chronic atrophic gastritis, Clostridioides difficile and Helicobacter pylori infection, peptic ulcer disease, celiac disease, small bowel bleeding, angiodysplasias, small bowel ulcers, inflammatory bowel disease (IBD), small intestinal bacterial overgrowth (SIBO), abdominal hernia, constipation, and diarrheal illnesses [141].

Decreased production of pepsin and hydrochloric acid limits the bioavailability of dietary and supplementary vitamin B12 [141,142]. Examples of bioavailability limitations include the use of acid suppressants to protect against medication side-effects (mainly NSAIDs), the presence of gastritis and/or duodenal inflammation, and esophageal disorders. Age-associated changes to intestinal epithelial cells and enterocyte function may result in insufficient nutrient absorption [143]. These limitations are also related to the higher prevalence of celiac disease in the elderly, reaching from 4 to about 25 percent [144]. In fact, this difference may be due to delayed diagnosis for celiac disease, mainly because of the atypical clinical manifestations of this enteropathy [141,143,145]. A higher incidence of hernias, adhesions, diverticulosis, and risk of obstruction may also contribute to bacterial overgrowth (SIBO) and chronic intestinal inflammation. Diverticular disease is rare in the general population, but it was found to affect 65% of people aged ≥65 years [141,146,147].

Abnormal hematological indicators such as inflammation-related normocytic anemia occur in approximately half of patients with hepatic cirrhosis [148]. Hepcidin plays a major role in hepatic disorders due to iron restriction. On the other hand, information regarding patients with cirrhosis is limited, and there is debate about the plasma erythropoietin (EPO) levels in these individuals. It is plausible that EPO elevation could be a result of renal hypoperfusion, hypoxia, anemia, or a hepato-protective and regenerative mechanism mediated by EPO. In contrast, inadequate EPO response in advanced cirrhosis might be attributed to poor hepatic synthesis capacity, decreasing co-factor levels, and inflammatory feedback mechanisms. Ultimately, the source of a potential increase in EPO production during certain stages of cirrhosis—whether from the kidney or liver—remains a lingering question [149].

While some changes associated with an aging GI system are physiologic, others are pathological and particularly more prevalent among those above 65 years of age [141]. Such GI diseases increase the risk of gastrointestinal bleeding.

Diseases of the gastrointestinal tract in old age are among the main causes of anemia due to the reduced absorption of micro- and macronutrients necessary for cell synthesis in erythropoiesis up to the erythrocytes themselves. It is challenging to unequivocally demonstrate anemia in the elderly as a result of chronic inflammation due to the overlapping causes of anemia such as disorders, impaired iron absorption, and the intake of other micronutrients.

### 4.5. Intestinal Dysbiosis

Our microbiota is subject to constant variation over our life course. The risk of intestinal dysbiosis (i.e., a significant reduction in beneficial microorganisms and an increase in opportunistic or pathobiont microbes in the gastrointestinal tract) is on the increase in old age depending on our health status, lifestyle, previous illnesses, and general inflammation [150,151]. Intestinal epithelial barrier dysfunction and increased permeability with aging, previously confirmed only in patients with inflammatory bowel diseases, have raised particular concerns. This is especially relevant for individuals with inflammatory bowel disease, nutritional deficiencies, overweight, metabolic syndrome, or those undergoing antibiotic therapy [152].

Josefsdottir et al. [153] demonstrated that the gut microbiota supports adequate hematopoiesis. Previous hypotheses suggested a signaling model involving the gut microbiome and the bone marrow. 

The gut microbiome–macrophage–iron axis recently discovered by Zhang and Gao [154] shows that microbiota-derived metabolites increase iron availability in a manner dependent on bone marrow macrophage erythrophagocytosis, which affects hematopoietic differentiation and blood regeneration. These include STAT1 signaling, type I IFN signaling in hematopoietic cells [155]. The presence of a favorable gut microbiota population ensuring adequate synthesis of short-chain fatty acids (SCFAs) such as acetate, butyrate, and propionate may contribute to erythrophagocytosis and favorable hematopoietic regeneration [156,157,158]. Soriano-Lerma and García-Burgos [159] reported a potential mediatory function of SCFAs in iron absorption, and possibly in anemia status modulation. A gradual increase in unfavorable intestinal microbiota contributes to an increased risk of intestinal absorption disorders in the elderly [143,160].

### 4.6. Autoimmune Diseases

The risk of chronic inflammation including autoimmunization increases with age. Paradoxically, an increase in the incidence of new autoimmune pathologies in all autoimmune diseases has not been recorded in older adults [161]. A recent population-based study by Conrad et al. [162] confirmed that they occurred almost twice as often in women as in men, and the mean age of diagnosis was 54 years. Only in entities such as Graves’ disease, pernicious anemia, and rheumatoid arthritis (RA) did the risk increase with age. For the other diseases (coeliac disease, inflammatory bowel disease and vasculitis), the incidence reached three different peaks: in childhood, early adulthood, and old age.

Even if we assume that the components of immunescence such as chronic inflammation, increased production of autoantibodies, and a decline in the immune response are most likely to be chronic autoimmunity rather than autoimmune diseases, they are still potential contributors to anemia in the elderly [163,164]. Some scientific reports support the link between anemia and autoimmune diseases, however, other causes of anemia should be considered such as anti-inflammatory drugs, glucocorticosteroids, biologic drugs that reduce iron-bioavailability and other essential micro- and macro-nutrients for erythropoiesis, or increased macrophage release [165]. This hypothesis may be supported by the decrease in Hb levels in RA, and the degree of clinical exacerbation of rheumatic disease. However, more research is needed into the relationships between anemia and autoimmunity and autoimmune diseases in older adults, taking into account confounding factors such as the use of medications that increase the risk of gastrointestinal diseases and limit bioavailability as well as other chronic diseases (e.g., CKD, hematological diseases, malnutrition, or hepatic diseases) [165,166,167,168,169].

Numerous research papers have reported that erythropoiesis-enhancing treatment reduced the severity of autoimmune diseases [170,171,172,173,174]. However, the conclusions should be treated with caution, as in some autoimmune diseases, EPO may produce pro-inflammatory or anti-inflammatory effects [175].

It is possible that the response of B and T lymphocytes in the autoimmunity process is potentially related to the increased risk of stress erythropoiesis, which does not allow older people to keep pace with the demand for erythrocytes [176,177].

## 5. Summary

Extensive studies on anemia in older adults have revealed significant outcomes including increased mortality, hospitalization rates, frailty, falls, mobility limitations, cognitive decline, dementia, functional dependence, and reduced quality of life. These findings are consistent across major cohort studies that excluded individuals with other conditions [6,178,179,180,181]. The multifactorial and highly prevalent nature of anemia in older adults is directly correlated with age. While the degree of anemia is mostly mild in the ambulatory setting, institutionalized patients exhibit higher rates of anemia of increased severity.

Aging-related changes are evident in the frequent occurrence of both anemia and indicators of inflammation in the elderly. It is crucial to recognize the interconnectedness of anemia and inflammaging, as they are, to some extent, manifestations of the same biological processes such as elevated levels of various proinflammatory mediators. Both conditions, anemia and inflammation, contribute to a heightened mortality risk, with the underlying causes of decreased survival likely stemming from a variety of factors. To enhance the clinical management of individual patients, a deeper understanding of the molecular mechanisms driving anemia and inflammation is essential. Our perspective emphasizes the need for personalized care based on the comprehensive clinical context when dealing with elderly patients.

A critical facet involves recalibrating reference values for older adults for key hematological parameters such as hemoglobin and hematocrit to align with the unique physiological changes associated with aging. This should make the diagnostic process more accurate and reflective of the health status of older individuals. Moreover, a diagnostic panel should be devised to encompass a spectrum of markers essential for comprehensive anemia assessment in older age. In addition to conventional indicators, special attention should be directed to hepcidin and inflammatory biomarkers such as CRP and cytokines such as IL-1β and TNF-α [52]. These indicators provide a more nuanced insight into the underlying causes of anemia, enabling tailored interventions that could address specific clinical conditions common in the elderly population.

Stratifying the elderly population into distinct age groups is another crucial modification proposal in the diagnostic approach. While anemia can affect individuals at various life stages, placing emphasis on those aged 65 and above recognizes the increased susceptibility to anemia-related issues in this demographic. Moreover, pinpointing high-risk groups such as individuals with specific chronic diseases ensures targeted and more frequent screenings for those who need it the most. Notably, persons aged 80 and above as well as institutionalized patients require even more vigilant monitoring, considering their advanced age and higher vulnerability to potential anemia-associated complications.

Moving beyond diagnostic measures, the multifaceted nature of combating anemia in the elderly necessitates the consideration of both non-pharmacological and pharmacological interventions. Non-pharmacological strategies, particularly dietary adjustments, have emerged as a cornerstone in anemia management. 

However, when pharmacological interventions are warranted, a judicious approach is essential. Considering the likelihood of elderly individuals already being on a myriad of medications, potential interactions must be carefully evaluated.

In essence, addressing anemia in the elderly requires a meticulous balance between comprehensive diagnostic measures and tailored interventions. By embracing these modifications in diagnostic testing and intervention strategies, health care professionals can navigate the complexities of anemia management in older individuals with greater precision and efficacy.

We ought to incorporate every accessible data found in the Clinical Practice Guidelines, which shall support the comprehensive management of these patients through a multidisciplinary and multimodal approach.

This review provides valuable insights that can guide future endeavors in refining the approach to anemia in the elderly, ultimately contributing to improved health care outcomes for this demographic population.

## Figures and Tables

**Table 1:** Selected studies examining the prevalence of anemia across various populations and its findings.

Study Name/Author	Country	Findings	Reference
EMPIRE study	Portugal	- higher prevalence of anemia in men than women (22.2% vs. 19.9%, respectively). - anemia prevalence increased with age, with the rates of 17.3% in the 65–79 age group and 31.4% in those aged 80 years and above.	[16]
Third US National Health and Nutrition Examination Survey (NHANES III)	USA	- a progressive increase in anemia prevalence with increasing age in the study participants aged ≥65 years, where the prevalence of 13% and 23% was recorded in subgroups aged 75–84 vs. ≥85, respectively. - data demonstrated that anemia was more prevalent in men than in women.	[1]
Zaninetti et al.	Italy	- anemia was prevalent in 62% of males aged ≥65 years compared to 44.1% prevalence in the group aged below 65 years. - similar observations pertained to female gender where the proportion of women ≥65 years with anemia reached 60.1% vs. 53.5% recorded in females below 65 years of age.	[17]
Muñoz et al.	Spain	- prevalence of anemia varied depending on the surgical intervention, ranging from 14% in prostate surgery to 61% in colorectal cancer cases.	[18]

**Table 2:** Examples of common diseases potentially leading to anemia in older adults.

Chronic inflammatory diseases	Pulmonary infectious Infective endocarditis Chronic urinary tract infection Chronic liver diseases Arthritis Chronic fungal infections COPD Chronic kidney disease Chronic heart failure Atherosclerosis
Autoimmune disorders	Rheumatoid arthritis Rheumatic fever Vasculitis Sarcoidosis
Hematological diseases	ICUS, CCUS, IDUS, CHIP, MDS Aplastic anemia
Cancer disease	Hematological malignancy, lymphomas, solid tumors
Endocrinological and metabolic diseases	Metabolic syndrome Overweight Sarcopenia Cachexia Malnutrition
Gastrointestinal diseases	Esophagitis, gastroenteritis, peptic ulcers, ulcerative colitis—GI bleeding Intestinal malabsorption syndrome (lactose, gluten intolerance) Celiac disease *Helicobacter pylori*, *Clostridioides difficile* Dysbiosis
Nutritional deficiency	B12, folic acid, vitamin D Amino acids Iron, zinc, selenium, copper,
Drug-induced anemia	Antibiotics, NSAIDs Proton pump inhibitors (PPIs), Chemotherapy Radiotherapy Polypharmacy

Abbreviations: CCUS, clonal cytopenia of unknown significance; CHIP, clonal hematopoiesis of indeterminate potential; COPD, chronic obstructive pulmonary disease; GI, gastrointestinal; ICUS, idiopathic cytopenia of undetermined significance; IDUS, idiopathic dysplasia of undetermined significance; MDS, myelodysplastic syndromes; NSAIDs, non-steroidal anti-inflammatory drugs.
